# Resolutions of Respect—American Academy of Dental Science

**Published:** 1898-03

**Authors:** 


### RESOLUTIONS OF RESPECT—AMERICAN ACADEMY OF DENTAL SCIENCE.

DR. THOMAS W. EVANS died suddenly of angina pectoris in Paris, November 14, 1897, aged seventy-four years. He was born in Philadelphia in 1823, and received in that dental centre, in his youth, theoretical training and practical experience in fine metallurgy and in dentistry. At an early age he was led by Dr. Brewster to go to Paris, and he there achieved one of the most brilliant careers which any dentist has ever accomplished. He was proficient in dental manipulations in the days when there were fewer skilled practitioners than there have been in recent years; and his other strong qualities enabled him to secure and retain as patients many of the most prominent persons of the world.

His services were not confined wholly to the routine of dentistry, but his abilities found channels for action in various hygienic, sanitary, and reform measures, and in a private diplomacy for which he was fitted by nature, and for the exercise of which he had unusual opportunities. In addition to his work as a brilliant practitioner his career was a marked illustration of the fact that the practice of our calling is not incompatible with high social position and the attainment of many honors through faithful service in other capacities. Born in America, and with American general and professional education, he kept up his acquaintance with our people and his friendship for our institutions. Embracing opportunities for lucrative investments, he became very wealthy; and it was his cherished design, whose fulfilment it is to be hoped no circumstances will frustrate, to devote a large part of his accumulations to the establishment of art and educational institutions in his native city.

Dr. Evans had been for many years an Honorary Fellow of the American Academy of Dental Science. In recognition of his high career and in testimony of our respect to his memory, we devote these pages of our records to this expression concerning him; and we would commend to our members the emulation of all that was worthy in his example, of thorough preparation for the life-work, of diligence in its pursuit, and of broad-spirited usefulness to the world as a man, a citizen, and a bestower of the rewards of his labors.

(Signed) CHARLES A. BRACKETT,
ROBERT R. ANDREWS,
EUGENE H. SMITH,
*Committee.*

